# Ad Hoc Modifications to a High Dependency Psychiatric Unit for People With Dementia During the COVID-19 Period

**DOI:** 10.2196/49618

**Published:** 2024-06-11

**Authors:** Thanita Pilunthanakul, Giles Ming Yee Tan

**Affiliations:** 1 MOH Holdings Pte Ltd Singapore Singapore; 2 Institute of Mental Health Singapore Singapore

**Keywords:** dementia, COVID-19, high dependency psychiatric unit, psychiatric intensive care unit, caregiver stress, SARS-CoV-2, psychiatric, psychiatry, mental health, health care system, Alzheimer, ward, care facility

## Abstract

The COVID-19 pandemic led to behavioral exacerbations in people with dementia. Increased hospitalizations and lack of bed availability in specialized dementia wards at a tertiary psychiatric hospital in Singapore resulted in lodging people with dementia in the High Dependency Psychiatric Unit (HDPCU). Customizations to create a dementia-friendly environment at the HDPCU included: (1) environmental modifications to facilitate orientation and engender familiarity; (2) person-centered care to promote attachment, inclusion, identity, occupation, and comfort; (3) risk management for delirium; and (4) training core competencies. Such practical solutions can also be implemented elsewhere to help overcome resource constraints and repurpose services to accommodate increasing populations of people living with dementia.

## Background

The spread of SARS-CoV-2 causing COVID-19 required significant reorganization of the Singapore health care system to support the rising number of COVID-19 cases and associated mental health issues [1,2]. Postponement of nonurgent clinic appointments due to reallocating hospital resources for pandemic control and patients’ fear of contracting COVID-19 led to significant delays in treatment and further increased the risk of psychiatric relapses [3]. Reduced physical and cognitive activity from the cessation of dementia daycare programs and limited social engagements due to restricted visits with friends and family further increased social isolation and behavioral exacerbations in people with dementia [4]. On top of this, unemployment, work-from-home policies, quarantine orders, and home-based learning put family members in closer contact with people with dementia. This struggle to cope with the increasing care demands of people with dementia has resulted in greater caregiver burnout and hospitalizations for respite care [5,6].

Due to the shortage of beds in the dementia wards at the Institute of Mental Health (IMH), a tertiary psychiatric care facility in Singapore, people with dementia are occasionally lodged in the High Dependency Psychiatric Care Unit (HDPCU) of the IMH, which was not initially designed for dementia-friendly interventions. In this viewpoint, we provide insight into easily implementable and practical creative solutions that have been used to accommodate people with dementia in the HDPCU during the COVID-19 pandemic, overcoming resource constraints and repurposing services in the face of changing needs using a patient-centered approach (summarized in Table 1).

**Table 1:** Summarized framework for customizing a care environment for people with dementia.

Principles	Examples
Environmental modifications to facilitate orientation and engender familiarity	Clear signs and signage Sizable and readable calendars and clocks Frequent reorientation by staff Nurse in areas allowing natural sunlight and views of greenery Simulate home-like surroundings, including partition areas to create “rooms” for different activities place photographs of friends/family close to the patient’s bed arrange regular communication between the patient and their family/friends via video calls
Person-centered care to promote attachment, inclusion, identity, occupation, and comfort	Obtain a detailed personal history from family/friends regarding the patient’s preferences Surround the patient with items that affirm their personhood, including playing songs that bring comfort or voice recordings of family/friends in the ward addressing patients by their usual/preferred nickname if possible, allow patients to wear/have sentimental items close by Empower patients to exercise choice as much as possible, no matter how small the decisions may be Encourage patients to join together for meals and games Engage patients in meaningful and mentally stimulating activities Express comforting interactions and validate patients’ concerns Patiently answer repeated questions and allow relatively more time to perform tasks
Risk management (delirium)	Actively take measures to prevent delirium, including restrict physical restraints to only when necessary, and even then, for the shortest duration required minimize medications that risk iatrogenic delirium Obtain a corroborative history regarding patients’ expression of discomfort to recognize signs of distress and address agitated behavior promptly
Core competencies (training if required) of staff	Understand core concepts of and practice person-centered care Geriatric-specific care, including fall and choking risks, along with activities of daily living support De-escalation skills for agitated geriatric patients

## Environmental Modifications of the HDPCU

The HDPCU is a specialized inpatient unit devised for patients with an acute psychiatric disorder linked to severe agitation or aggression, placing them at significant risk to themselves or others, leading to the requirement of close monitoring. The nursing counter is sandwiched between two locked gender-specific cubicles with 4 and 6 beds, respectively, and single bathrooms. The nurse has a full view of both cubicles and there are various discreetly placed security cameras. Items that could fuel self-harming or suicidal behaviors, such as wires for electronics, plastic bags, detergents, sharp pencils, and utensils, are strictly prohibited in the ward. There is a 2:1 nurse-to-patient ratio. Staff are specially trained in swift de-escalation to ensure safety and prevent violence, including applying physical restraints and administering oral and intramuscular sedation if required. A psychiatrist, a junior doctor, and the nursing and allied health care team are on site to manage the patients.

People with dementia often experience disorientating situations due to separation from familiar settings, people, and routines. Dementia wards have specific modifications to orient people with dementia, such as legible signage; large clocks; brighter lighting; and contrasting-colored walls, furniture, and utensils [7]. Renovating the HDPCU to suit such requirements was not immediately feasible. Hence, modifying the environment to have clear signs indicating the bathrooms; a sizeable hand-drawn daily calendar facing the bed indicating the date, day, month, and year; and verbal reorientation 3 times a day were implemented to facilitate orientation. People with dementia were also nursed opposite a readable digital clock and beside big windows that offered a view of greenery and allowed in natural sunlight.

Dementia wards engender familiarity by creating homey surroundings, including paintings hung along corridors and divided kitchen, bedroom, and living room spaces. Since safety is of utmost priority in the HDPCU, rules are often strict, and cubicles are designed to be relatively smaller than found in other wards along with an open layout for easy monitoring. These restrictions may cause people with dementia, particularly those who like to wander, to feel trapped and anxious. Given the nature of patients admitted to the HDPCU, the noisy and disruptive atmosphere can destabilize and frighten people with dementia. To create a calm environment that minimizes overstimulation and distractions, people with dementia were nursed in a partitioned visitors’ area accessed via a corridor adjacent to the cubicles and nursing counter. Families of people with dementia were encouraged to bring photographs to place in front of patients’ beds and participate in regular video calls from the ward smartphone to lessen the effects of visitor restrictions during the pandemic [8]. The sectioned area also reduced the risk of impaired sleep-wake cycles and sun-downing behaviors that are common among people with dementia and could provoke other patients. The improvised space simulated a private bedroom, while a wheel-in television and movable couches in the cubicles’ shared living area imitated a makeshift living room.

## Promoting Person-Centered Care

In person-centered care for people with dementia, personhood consists of attachment, inclusion, identity, occupation, and comfort. Emotional distress is usually triggered by unmet needs related to aspects of personhood. Obtaining a detailed personal history from the family regarding the preferences of people with dementia is essential to affirm personhood. For one such patient, playing Chinese songs from his childhood, hearing voice recordings of family, addressing him by his preferred nickname, and wearing a jacket gifted from his daughter in the ward provided a sense of comfort, identity, and continuation of self. Empowering the patient to exercise choice as much as possible, even for tasks as small as choosing a preferred snack, preserves autonomy and dignity. Encouraging people with dementia to join other patients during meals and games instills a sense of inclusion and occupation [9,10]. Engaging people with dementia in meaningful activities mentally stimulates and reduces the restlessness related to the tendency to worry about their situation. Expressing warmth through comforting interactions, patiently answering repeated questions, allowing them more time to perform tasks, and validating concerns can help to settle the wariness and diminished sense of attachment experienced by these patients. These efforts promote the therapeutic relationship and trust between staff and people with dementia, ultimately reducing aggression and distress.

## Risk Management

Lastly, because people with dementia are prone to delirium during acute hospitalization, the HDPCU team actively took measures to prevent this risk. Physical restraints were only applied if verbal de-escalation repeatedly failed and the extreme agitation posed a safety risk to themselves or others; when required, patients were restrained for the shortest duration necessary. Wherever possible, medications that risk iatrogenic delirium in people with dementia were avoided, such as short-acting benzodiazepines for tranquilization, anticholinergic drugs, and opioid-containing analgesics. People with dementia often have issues communicating their needs and are likely to only respond to their present state due to verbal difficulties and memory problems. Obtaining a further history regarding the patients’ typical behavioral patterns and expression of discomfort from pain, hunger, thirst, or constipation helped the team promptly recognize signs of distress and address agitated behaviors early without escalating to restraints.

## Prospects

In conclusion, hospital care conditions can be difficult for people with dementia as they require familiarity, frequent orientation, and a high level of staff trained to handle their needs. The HDPCU adapted to rapid hospital protocol and health care policy changes during the COVID-19 pandemic and the resultant rise in the inpatient dementia population. Although the HDPCU staff were not geriatric-trained, the favorable staffing ratio and expertise in handling agitated and aggressive patients made it easier to implement person-centered care. Such conditions may not be available in nonspecialized wards, posing a challenge for catering to the increasing population of people with dementia admitted to hospitals in Singapore. Nonetheless, creative solutions could be established to customize the environment for such patients aptly. Hospitals could also consider bringing in key “experts” such as psychogeriatricians and geriatric nurses to advise on optimizing nonspecialized wards and provide training to care for people with dementia.

## Abbreviations

HDPCU: High Dependency Psychiatric Unit
IMH: Institute of Mental Health
